# Turning promise into practice: Crop biotechnology for increasing genetic diversity and climate resilience

**DOI:** 10.1371/journal.pbio.3001716

**Published:** 2022-07-26

**Authors:** Sarah Garland, Helen Anne Curry

**Affiliations:** 1 The Earth Institute, Columbia University, New York, New York, United States of America; 2 Department of History and Philosophy of Science, University of Cambridge, Cambridge, United Kingdom

## Abstract

As climate change increasingly threatens agricultural production, expanding genetic diversity in crops is an important strategy for climate resilience in many agricultural contexts. In this Essay, we explore the potential of crop biotechnology to contribute to this diversification, especially in industrialized systems, by using historical perspectives to frame the current dialogue surrounding recent innovations in gene editing. We unearth comments about the possibility of enhancing crop diversity made by ambitious scientists in the early days of recombinant DNA and follow the implementation of this technology, which has not generated the diversification some anticipated. We then turn to recent claims about the promise of gene editing tools with respect to this same goal. We encourage researchers and other stakeholders to engage in activities beyond the laboratory if they hope to see what is technologically possible translated into practice at this critical point in agricultural transformation.

## Abstract 2

Will gene editing contribute to improved crop diversity and climate resilience? In this Essay, the authors look at lessons from past biotechnology efforts to inform action for the future.

In 1970, a virulent fungal blight decimated the United States corn harvest. This southern corn leaf blight epidemic was linked to a subset of genes that made certain varieties more susceptible than others—genes that also happened to be shared across some 75% of commercial varieties [1,2]. The blight arrived just as scientists concerned about a more general loss of genetic diversity in crop plants, both in the US and abroad, were finally gaining the ear of governments and philanthropies. They called for more and better gene bank facilities and, brandishing blighted maize as the canary in the coal mine, a re-diversification of industrial crops [3–5].

With these concerns as motivation, some researchers pointed to the possibility of increasing genetic diversity among cultivars of a given crop with a brand-new biotechnology: recombinant DNA [6]. These techniques could be used to introduce novel genes into the high yielding but genetically narrow lines dominating commercial markets. But this anticipated use of recombinant DNA technologies for expanding genetic diversity has yet to materialize.

The need to diversify crops is coming back into focus due to increasingly urgent climate and nutrition challenges [7–9]. Diversified agricultural systems are more resilient to climate hazards and can stabilize food production [10]. Increasing genetic diversity, by both widening the genetic bases of commonly cultivated crop species and restoring a greater number of species to cultivation, is therefore a high priority for climate action.

Biotechnology is once again offering a path forward. Today’s plant scientists are developing gene editing techniques that could facilitate genetic diversification of commodities like wheat, rice, and maize and potentially support the adoption or continued cultivation of “neglected” crops that have been less often subject to crop breeding and development activities. But will gene editing really generate a diversity boom? Can it upend a pattern of genetic narrowing that breeders and botanists have observed since the late 19th century—a pattern frequently pinpointed as a major source of vulnerability in global agricultural production systems?

Excavating comments that reveal an often forgotten subset of early aspirations for recombinant DNA technologies provides insight on contemporary dialogue about gene editing. The history of these technologies illustrates the extent to which diversification depends on much more than a laboratory toolkit. Awareness of past efforts can inform today’s aspirations for and decision-making about the use of crop biotechnologies to enhance genetic diversity.

## Intervening in evolution: The emergence of recombinant DNA

The turn of the 20th century saw the emergence and rapid development of genetics as a research discipline and with it the celebration of specific knowledge and tools as novel means of controlling the heredity of plants, animals, and humans. The “rediscovery” of Gregor Mendel’s studies of inheritance in peas in 1900 provided ambitious discipline builders with a simple framework for explaining the transmission of traits and enabled many to recruit further resources for research [11,12]. Although the agenda set for genetics research varied from one national context to the next, it was often closely allied with agricultural work and benefitted from the expectation that geneticists’ expertise would allow them to breed new crop varieties with untold efficiency and precision [13,14].

**Fig 1:**
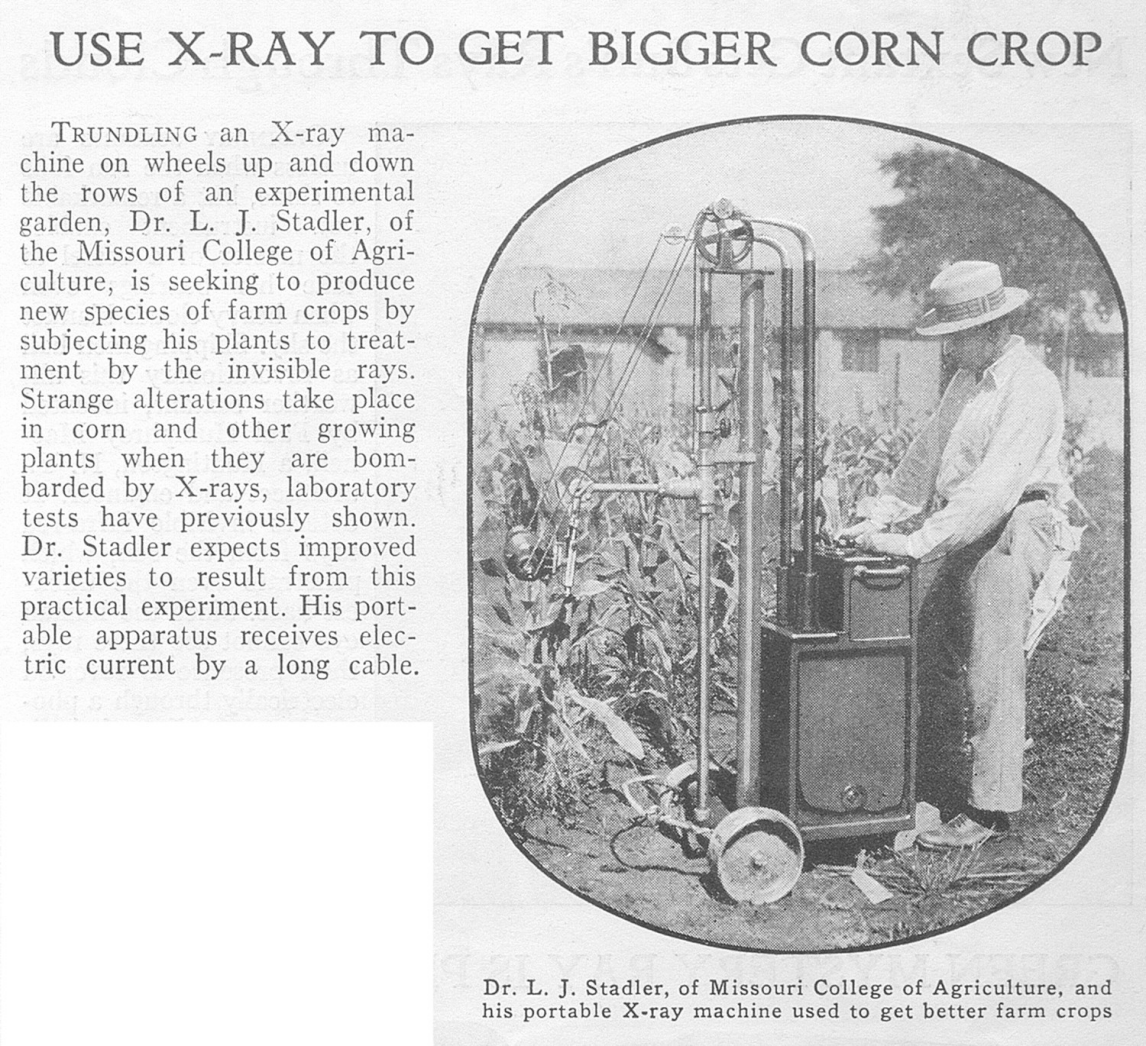
The geneticist Lewis Stadler X-rays corn in hopes of inducing mutations. From *Popular Science Monthly*, January 1932, 47.

Early breeders-turned-geneticists typically deployed the well-established approaches of hybridization and selection in their efforts to make new varieties “to order” and were able to generate and market new seed products as a result. Many were nonetheless already experimenting with tools that allowed direct physical manipulation of genes and chromosomes by the 1910s, hoping that these would open further horizons for plant breeding [15]. Exposing seeds, bulbs, and buds to X-ray tubes, chemical mutagens, and radioisotopes prompted genetic changes that scientists of the 1940s and 1950s frequently characterized as “accelerating evolution” [16]. They also often hinted that bigger, heartier, more profitable crops lay just ahead (Fig 1). Yet despite big hopes—and bigger hype—mutagenic methods delivered only modestly on researchers’ bold claims.

Then a new set of tools arrived, technologies allowing genes from one organism to be inserted into the genome of another, even across species boundaries. Where the “mutation breeders” of the mid-20th century envisioned speeding up evolution, some molecular biologists of the 1970s saw themselves abandoning evolution altogether as they generated “transgenic” organisms [17]. Crop breeders would no longer be limited to the gene combinations available within established varieties, landraces, or even species and could transfer genes (and therefore traits) far more widely than ever before (Box 1). Some believed they could also tackle a problem understood to that point as an inevitable by-product of plant breeding: the loss of genetic diversity.

**Box 1. Glossary**

**Crop domestication**

Processes by which the reproduction of a plant species comes to be under the control of human groups, typically so that it can be used for food, clothing, shelter, or other purposes.

**Crop wild relative**

A plant species closely related to a domesticated agricultural crop, for example, an evolutionary ancestor or related taxon.

**Landrace**

A locally adapted plant variety or animal breed maintained as a distinct population.

**Neglected crop**

A crop species that has not been subject to significant research for commercial development and is therefore potentially underused in agriculture.

**Traditional breeding**

Methods that rely on crossing together plants via sexual reproduction.

**Mutation breeding**

Techniques that use chemical or radiation treatment to induce random changes to a plant’s DNA.

**Recombinant DNA**

Molecular biology techniques that facilitate combining genetic material from multiple sources.

**Transgenesis**

The insertion of DNA from one organism into the genome of another unrelated organism. For crops, this involves isolating genetic material from an organism like a bacterium or a different plant species and transferring it into the crop genome.

**Cisgenesis**

Similar to transgenesis, but instead of the inserted DNA coming from an unrelated organism, it originates in a related plant, such as a crop wild relative.

**Gene editing**

A range of techniques that induce targeted genetic changes. Many products generated via gene editing only contain mutations in the crop plant’s own genome instead of insertion of DNA from an external source.

For almost a hundred years, experts had insisted that as farmers adopted increasingly uniform “modern” varieties produced by professional breeders, the more heterogeneous varieties previously maintained by farmers would vanish [18,19]. And research has suggested that, for nearly as long, breeders’ efforts to produce distinct, uniform, and stable crop varieties has been associated with a narrowing of genetic diversity [19,20], even if farmers’ varieties rarely disappear completely [21]. By the mid-20th century, recognition of this pattern had led to the creation of cold-storage facilities dedicated to extending the lifespan of seeds in several countries. These seed bank facilities were tasked with keeping extant the genetic diversity that was believed to be disappearing from fields [22–24].

The loss of genetic diversity associated with the transition from varied and genetically heterogeneous farmers’ varieties to breeders’ more standardized products at ever-increasing scales became a crisis scenario for many botanists and crop scientists in the 1970s, and not just because of the US corn blight. A Green Revolution in agricultural production, in which farmers of South Asia, Latin America, and the Middle East were thought to be transitioning en masse to “modern” varieties, was first declared in 1968 and swiftly identified as a potential diversity bottleneck [25].

Green Revolution varieties, such as the semidwarf wheat first developed in Mexico and the semidwarf rice IR-8 bred in the Philippines, had complex genetic origins and were celebrated in particular for their ability to survive in diverse ecological contexts given the right inputs. The dissemination of these new varieties, along with increased availability of nitrogen fertilizers, irrigation, and other inputs, led to significant increases in grain production in many places and to a drop in food prices [26].

As many observers noted, then and since, these gains in yield were accompanied by several social and environmental costs. One consequence of the widespread adoption of these “miracle varieties” [27,28] was an unprecedented shift toward uniformity in the genetic composition of farmers’ fields. This rapid conversion led to urgent calls to conserve diverse landraces [18,25].

Meanwhile, global population growth and concerns about resource scarcity, factors that had spurred the crop research associated with the Green Revolution, also focused international attention on many other kinds of biological diversity. The loss of this diversity, whether represented in genes, species, or ecosystems, spurred new conservation activities [29].

Set in this context, circumventing evolution through transgenic engineering was celebrated by some plant scientists as a contribution to conserving or even amplifying genetic diversity, especially in industrial crops. Ambitious researchers, along with eager agricultural administrators and policy-makers, imagined screening the world’s biodiversity, identifying genes of interest, and transferring them into valuable lines. They hoped the result would be an unprecedented mobilization of genetic material to create diversity not known or even imaginable in nature [6].

Although enhancing genetic diversity in industrial crops was never the most prominent goal sought by researchers working with recombinant DNA in crop development, this potential outcome surfaced often in conference proceedings and other forums where technical workers came together to project the future of their field. Among the most frequently promised diversifications were grains with enhanced protein content, an expanded range of agricultural crops able to fix nitrogen, and modern cultivars with the traits for heat- and drought-resistance possessed by their wild and weedy relatives or landraces stored ex situ in seed and gene bank collections [6,30–32].

Recombinant DNA techniques were sometimes touted not only as making seed and gene bank collections more valuable, facilitating breeders’ use of stored genetic diversity, but also as instruments for focusing public attention on the importance of biodiversity conservation [33]. The motivation was not shared by all biotechnologists, but some scientists hoped that a public dazzled by genetic engineering “breakthroughs” that mobilized genetic biodiversity in new ways would be more inclined toward conservation activities [34].

## Unfulfilled promise: Transgenic crops in the field

In retrospect, these hopes that transgenic tools would be a boon for biodiversity seem misguided. The vast majority of transgenic crop varieties cultivated today possess only 2 engineered traits. They either contain 1 or several genes derived from *Bacillus thuringiensis* (Bt) that result in insect-resistance [35] or they contain 1 or several genes also of microbial origin that confer tolerance to herbicides, chiefly glyphosate [36]. Increasingly, they are engineered to include both Bt insect-resistance and herbicide-tolerance (HT) [37]. In 2019, these 2 traits accounted for more than 99% of global acreage planted with transgenic varieties [38].

The deployment of these crops has been controversial, and researchers are continually exploring whether and under what circumstances their use enhances agricultural productivity and sustainability [39–42]. One thing is clear, however, transgenic tools have not expanded crop diversity in the ways that some imagined at the emergence of the technology. If anything, the spread of a few genes linked to just 2 traits has provoked a novel kind of genetic homogeneity in industrial crops that is also unprecedented in scale. The genes implicated in the southern corn leaf blight, which were derived from a single progenitor plant and encoded a form of male sterility useful in seed production, became sources of vulnerability because they had proliferated so extensively across US maize varieties. Today, the Bt transgenes are even more ubiquitous, found not only across countries but also across crop species, too [35,43,44].

Some transgenic crops possess traits other than herbicide tolerance and Bt. One example is Golden Rice, generated as a tool to address the public health concern of Vitamin A deficiency that affects primarily women and children. Through the incorporation of 2 genes from other species, scientists were able to enrich the rice grains with beta-carotene, a precursor of Vitamin A [45]. In development since 2001, the commercial production of Golden Rice has been long delayed due to many compounding factors including regulation, resistance to transgenic crops, and misapprehension of the cultures of rice cultivation and consumption in target regions [46–48]. The Philippines, a country where many are affected by Vitamin A deficiency, announced that 2022 will be the first year that Golden Rice seeds will be mass produced for cultivation by Filipino farmers [49].

Another transgenic crop in cultivation is the Rainbow papaya, which resists the devastating papaya ringspot virus thanks to insertion of a gene from a weak strain of the virus itself [50,51]. These were released to Hawaiian growers in 1998. By 1999, 50% of the area used for commercial papaya in Hawaii was planted with transgenic papaya, saving the Hawaiian papaya industry [52].

Further traits are in the pipeline, many of which are derived from wild relatives of crop plants. The Cavendish banana makes up almost 50% of the world’s banana production [53] and is dangerously susceptible to the disease Fusarium wilt tropical race 4 (TR4), which does not respond to chemical control. Researchers have generated Cavendish bananas resistant to TR4 by inserting a disease resistance gene from a wild banana [54].

Meanwhile, in the United Kingdom, scientists have introduced 3 genes from potato relatives into a cultivated variety to confer resistance to late blight and tuber blight—diseases notoriously catastrophic for growers. It is expected that the stacking of these multiple wild resistance genes will provide more robust disease resistance than previous conventional breeding efforts because it will be more difficult for the pathogen to adapt to the plants’ defenses [55,56].

Even considering these efforts, there is an undeniable disconnect between what was imagined with respect to diversifying agricultural crops through genetic engineering and the reality that unfolded. For many reasons (discussed further below), the opportunities created by recombinant DNA technology with respect to crop diversity remain unfulfilled in practice.

## A new hope: Gene editing for crop diversity

Leading plant scientists today praise innovative gene editing techniques as game-changing methods destined to fulfill aspirations for expanding crop genetic diversity through biotechnology [57–60]. This fanfare sounds familiar, as scientists throughout the history of crop breeding have heralded various innovations in similar ways, most recently with the expectation that recombinant DNA would create paradigm-shifting possibilities (Table 1). What, if anything, is different about the potential of gene editing technologies with respect to genetic diversity?

**Table 1:** What were scientists saying?

Example quotations from leading scientists on the potential for emerging crop breeding techniques to expand genetic diversity	Year	Method referenced
It is a comparatively easy matter to produce any desired breed of animals or any desired variety of plants when the various characters desired can be found scattered in breeds or varieties that can be cross bred. Application of these principles [of mendelian inheritance] will undoubtedly play an important part in the improvements of farm crops and farm animals in the future [61].	1911	Hybridization and selection informed by mendelian genetics
The study of [genetic] mutations, and, through them, of the genes themselves, has heretofore been very seriously hampered by the extreme infrequency of their occurrence under ordinary conditions, and by the general unsuccessfulness of attempts to modify decidedly, and in a sure and detectable way, this sluggish “natural” mutation rate. Those working along classical genetic lines may be drawn to the opportunity, afforded them by the use of X-rays, of creating in their chosen organisms a series of artificial races, it should be possible to produce, “to order,” enough mutations to furnish respectable genetic maps. Similarly, for the practical breeder, it is hoped that the method will ultimately prove useful [62].	1927	X-ray radiation-induced mutation breeding
The ability to induce chromosome doubling [with application of a colchicine solution], therefore, is of importance to practical as well as to theoretical genetics. With increasing knowledge of the constitution of chromosomes and methods whereby their structure and behavior may be altered, there arises an opportunity for the genetics engineer who will apply knowledge of chromosomes to building up to specification forms of plants adapted to the surroundings in which they are to grow and suited to specific economic needs [63].	1937	Colchicine chemical-induced mutation breeding
Genetically superior plants derived from modern crop improvement programs typically require a high level of crop management. Included in a management regime may be the input of increasingly expensive nitrogen fertilizers as well as the extensive use of pesticides and herbicides, all of which can result in toxic residue accumulation in the environment. In addition, the high degree of inbreeding and the narrowing of the genetic base of widely cultivated crops cause increasing concern about the susceptibility of crops to major disease outbreaks and imply that important genetic traits may be lost as world germplasm is reduced. With problems such as these, it is not surprising that the advent of recombinant DNA technology is generating excitement. A whole range of very specific plant genetic modifications can now be considered, with the use of methods that may someday generate a genetic diversity not naturally present in cultivated plants [6].	1983	Recombinant DNA
The genetic bottlenecks imposed on our modern crops by the long domestication process have removed most of the genetic diversity available for breeding, which makes further improvement of elite varieties by traditional breeding technology a cumbersome process. CRISPR/Cas-based new breeding tools including multiplex editing, fine-tuning of gene expression, and de novo domestication now provide plant breeders with exciting new opportunities to generate genetic diversity for breeding in an unprecedented way [58].	2019	CRISPR-Cas9 gene editing

One key difference between gene editing and recombinant DNA lies in the nature of the genetic diversity being explored. Instead of mining gene bank collections or searching the genomes of other organisms, many efforts to expand genetic diversity with gene editing focus on “unlocking” the variation within a plant’s own genome. Whereas early recombinant DNA programs intended to defy evolution, these gene editing goals are reminiscent of the hopes of the mid-20th century mutation breeders: accelerating evolution. This time, however, instead of randomly generating variation with radiation treatment or mutagenic chemicals, the technology is able to deliver more precise and predictable genetic change [64,65].

Gene editing methods employing site-specific nucleases (ZFNs, TALENs, CRISPR-Cas9) are guided either by protein engineering or RNA sequence complementarity to target a specific location in an organism’s genome. These gene editing techniques, combined with the rapid advance of genetic sequencing technologies and digital analysis tools, can potentially be powerful instruments for increasing crop genetic diversity. A brief overview of several strategies that researchers currently imagine using to achieve this end indicates the range of possibilities.

One strategy to increase genetic diversity using gene editing is to alter genes that confer undesirable traits, such as susceptibility to disease. For example, disrupting genes involved in plant susceptibility to powdery mildew in crops such as tomato, grapevine, and wheat induced resistance to the disease [66–68].

In other cases, a sought-after trait is not associated with loss of a gene’s function but rather a change in the extent to which a certain gene is expressed. Gene editing can address these traits, too. Instead of targeting coding sequences, as in the case of powdery mildew, the editing can be directed to genetic regulatory elements, thereby altering the level of gene expression [58,69]. This technique has been demonstrated in tomato, for which the expression levels of certain development genes determine how much the plant branches. Moderate branching can lead to increased flowering and yield while too much branching is associated with low fertility. By combining natural and gene-edited mutations in regulatory elements of these key genes, researchers were able to control their “dosage,” conferring an optimal level of branching [70].

Gene editing also offers opportunities to radically rethink the breeding process in ways that enhance genetic diversity by “restarting” crop domestication. Crop domestication relies upon a combination of spontaneously occurring genetic mutations and artificial selection by humans. In wild rice, for example, grains shatter in order to widely disperse the seed. During rice domestication, a mutation arose that caused non-shattering grains, a trait beneficial for early agricultural societies and therefore selected for cultivation. Rice wild relatives today carry beneficial traits like adaptation to diverse growth environments but their grains still shatter.

“De novo domestication” uses gene editing to generate mutations that are known to be markers of domestication in crop wild relatives or neglected crops. This “restarts” the domestication process, with the aim of reclaiming beneficial traits retained by these plants such as nutrient content or stress tolerance, while also enabling essential domesticated traits—like non-shattering grains. The technique has already been demonstrated in multiple plant species, including tomato, rice, and the neglected crop ground cherry [71–74]. Editing key genes for de novo domestication holds promise for increasing genetic diversity, but to successfully implement this process in crops that reach cultivation will likely require further development after edited plants are produced, including subsequent breeding to make crops suitable for particular ecological and cultural contexts [75].

Chromosome engineering via gene editing presents even more possibilities for increasing genetic variation [76–78]. The phenomenon of “linkage drag,” in which detrimental traits travel with desired ones due to their position on the chromosome, frequently stymies efficient conventional breeding. Strategic DNA breaks generated via gene editing can release this genetic linkage, producing new chromosome arrangements that can liberate beneficial genetic material from unfavorable associations. Scientists can also use gene editing to invert chromosome regions to modify recombination frequency. Depending on the design of the inversion, the method can either unleash variation to discover new traits or lock in desired traits so they are not lost as breeding continues.

As a final illustration, in an approach that seems to materialize the ambitions of mutation breeders past, gene editing can be used as a tool for “directed evolution,” a technique designed to accelerate the evolution of a specific protein. First, scientists create CRISPR libraries targeting every possible location in the gene of interest. The goal is to create random variation in the gene in the hopes of finding a mutation that leads to an optimized version of the gene’s protein product. After performing the gene editing step, mutants are screened for the desired enhanced phenotype [79,80].

Various technical obstacles to efficient gene editing in plants remain, like overcoming the bottleneck of plant tissue culture [81]. But methods are rapidly advancing. It would be reasonable to predict a future boost in genetic diversity because of gene editing if these technologies are deployed as scientists currently imagine. The technological potential today exists—and yet, recalling the promises made of recombinant DNA techniques in the 1970s and 80s, there is reason to wonder whether this potential can be realized in practice [82].

## Looking ahead: Engagement beyond the laboratory

Historically, plant breeding in the hands of professional breeders, whether funded by state governments or private industry, has trended in the direction of narrowing crop diversity [19]. As 20th century history shows, even when novel biotechnologies promised great genetic diversification, they did not deliver. What happened between lab and field that explains how technologies that promised so much fell short?

In the case of recombinant DNA technology, this question has been the subject of innumerable studies and much debate [83,84]. Issues including (but not limited to) biological challenges, strong intellectual property protections, seed industry privatization and consolidation, regulatory obstacles, activist protest, consumer distrust, and limited corporate visions have all played a role in constraining the crop varieties developed based on recombinant technology [48,85–90]. It is not the intention of this Essay to revisit the complex historical trajectories of transgenic crops in different farming contexts around the world. What is relevant for this discussion is the observation that most—though not all—of these factors extend beyond the laboratory. It is therefore reasonable to assume that, despite the technological advances of gene editing and the avenues they might open for increasing crop genetic diversity, it is unlikely that the imagined possibilities will come to fruition without fundamental shifts in many domains.

There is good reason to advocate for such changes, as the urgency of expanding crop genetic diversity has only intensified since the 1970s. From tolerating drought to fighting off pests to enabling healthy diets, increasing crop genetic diversity is an important strategy for ensuring climate resilience and meeting nutritional needs in many different farming contexts—from subsistence cultivation to industrial monocrop production [7,91]. Not all diversification strategies include biotechnology [92] and facilitating the use of biotechnology—whether gene editing, recombinant DNA, or other tools yet to come—does not have to exclude other methods. Simultaneously tackling climate change and hunger in the near term will require a wide range of approaches to enhancing genetic diversity, and in some contexts will likely include the responsible use of crop biotechnology [93,94].

Yet history shows that laboratory achievements do not lead to promised crop diversification when other elements of agricultural research and production systems do not facilitate it. Do financial and institutional resources for research support crop development for diversity? Is funding in place to support projects that may not represent immediate economic gain? Are crop biotechnology regulations designed to promote development of diverse traits? Do varietal registration requirements encourage genetic diversity in varieties? Do farmers know how to manage a new variety? Are processors prepared to handle a different product? Are consumers ready to eat foods prepared from reintroduced “neglected” crops? All of these concerns arise in addition to the more often asked question of whether a particular community is willing to eat transgenic or gene-edited foods. An ever-growing number of tools to diversify crops are unlikely to become significant if they lack a pipeline that also facilitates diversification or fail to address the needs of growers and eaters in a wide variety of ecological and cultural contexts.

It is tempting to view these considerations as factors “downstream” of the laboratory. Yet they are inextricably linked to all stages of applied research, from project funding to commercialization to social and environmental impact. Consider the 2018 European Court of Justice ruling that gene-edited products would be strictly regulated in the European Union [95]. This decision not only had direct implications for commercialization but also was expected to “squeeze science” [96] due to waning interest in financially supporting research advances subject to such restrictions in practice. After the controversial ruling, the European Commission embarked on a series of studies and consultations to determine whether their existing regulations are still suitable, and the future of gene editing in the European Union is yet undecided [97,98].

Crop biotechnologists hoping to see their diversity-enhancing lines flourish in fields must therefore be ready to engage in discussions beyond laboratory walls. To forge a path forward, researchers can raise critical awareness and foster shared support among sectors for increasing crop genetic diversity to tackle urgent global challenges.

Part of this effort will include clearly illustrating how biotechnology can play a meaningful role in this transformation. But researchers’ claims about what new biotechnologies can achieve must account for the inseparability of the technical and the sociopolitical. Past failures to recognize that outcomes are largely shaped by the expectations and decisions of farmers, consumers, and policymakers has led to erosion of public trust that continues to affect the acceptance of crop biotechnology [99,100].

Designing tools that enable researchers to understand and assess the effects of their efforts on desired agricultural transformations may aid in raising awareness [101]. This work will require closer engagement with diverse farming communities to better understand local and regional ecosystems, preferred cultivation practices, and distinct cultural needs. Moving toward climate-resilient agricultural production calls for context-specific interventions rather than universal solutions.

Using biotechnology to expand crop genetic diversity will also require that researchers understand the many junctures in crop variety development and dissemination, especially those linked to seed commercialization, that work against such expansion [19]. Addressing these obstacles will involve addressing issues as varied as farmer seed choice, seed certification processes, and international intellectual property regimes. It will require engaging with and developing further interdisciplinary and participatory research efforts to map infrastructural obstacles and to indicate actions that different stakeholders can take to facilitate genetic diversification.

Overcoming the long association of professional plant breeding with narrowing genetic diversity and the more recent restriction of biotechnological tools to the same fate is a daunting challenge. Farming systems that rely on commercial varieties produced by professional breeders will always see a genetic narrowing at some scale. But there are opportunities to remove or mitigate points at which significant bottlenecks occur even in these systems. Several scientists and research institutions are already paving the way forward. The OpenPlant initiative [102] in the UK is one such program; it strives to create open-access plant synthetic biology tools, eliminating barriers to innovation, and incorporating the need for technology transfer into the fabric of applied research.

This program’s focus on reducing intellectual property protections and therefore economic barriers to plant biotechnologies recognizes that the objectives embodied in new technologies, including crop varieties, typically reflect the goals of those holding the purse strings for technical development. The history reviewed above, from the Green Revolution of the 1960s to the gene revolution that followed, shows how multinational corporations and select philanthropies have played an outsized role in directing crop development in the past 70 years, and that this pattern of investment has not been conducive to producing or even sustaining diversity. Moving forward, the major actors involved and the priorities they set will determine how crop biotechnologies are used, and ultimately, what crops farmers choose to grow.

For crop biotechnology to help achieve ambitious climate and nutrition goals, action needs to happen now. Researchers, farmers, industry leaders, policy-makers, and the public are all responsible for creating circumstances that promote greater crop diversity, instead of reinforcing patterns that extend uniformity. These efforts, technical and social, are essential to ensuring that promising biotechnology discoveries are effectively implemented to drive sustainable and equitable agriculture systems in the face of a rapidly changing climate.
